# The Diagnosis of Urinary Tract Infection in Young Children (DUTY) Study Clinical Rule: Economic Evaluation

**DOI:** 10.1016/j.jval.2017.01.003

**Published:** 2017-04

**Authors:** William Hollingworth, John Busby, Christopher C. Butler, Kathryn O’Brien, Jonathan A.C. Sterne, Kerenza Hood, Paul Little, Michael Lawton, Kate Birnie, Emma Thomas-Jones, Kim Harman, Alastair D. Hay, on behalf of the DUTY Study Team

**Affiliations:** 1 School of Social and Community Medicine, University of Bristol, Bristol, UK; 2 Nuffield Department of Primary Care Health Sciences, University of Oxford, Oxford, UK; 3 Division of Population Medicine, Cardiff University, Cardiff, UK; 4 South East Wales Trials Unit (SEWTU Centre for Trials Research), Cardiff University, Cardiff, UK; 5 Primary Care and Population Sciences Division, University of Southampton, Southampton, UK; 6 Centre for Academic Primary Care, NIHR School of Primary Care Research, School of Social and Community Medicine, University of Bristol, Bristol, UK

**Keywords:** antibacterial agents, diagnosis, economics, medical, pediatrics, urinary tract infections

## Abstract

**Objective:**

To estimate the cost-effectiveness of a two-step clinical rule using symptoms, signs and dipstick testing to guide the diagnosis and antibiotic treatment of urinary tract infection (UTI) in acutely unwell young children presenting to primary care.

**Methods:**

Decision analytic model synthesising data from a multicentre, prospective cohort study (DUTY) and the wider literature to estimate the short-term and lifetime costs and healthcare outcomes (symptomatic days, recurrent UTI, quality adjusted life years) of eight diagnostic strategies. We compared GP clinical judgement with three strategies based on a ‘coefficient score’ combining seven symptoms and signs independently associated with UTI and four strategies based on weighted scores according to the presence/absence of five symptoms and signs. We compared dipstick testing versus laboratory culture in children at intermediate risk of UTI.

**Results:**

Sampling, culture and antibiotic costs were lowest in high-specificity DUTY strategies (£1.22 and £1.08) compared to clinical judgement (£1.99). These strategies also approximately halved urine sampling (4.8% versus 9.1% in clinical judgement) without reducing sensitivity (58.2% versus 56.4%). Outcomes were very similar across all diagnostic strategies. High-specificity DUTY strategies were more cost-effective than clinical judgement in the short- (iNMB = £0.78 and £0.84) and long-term (iNMB =£2.31 and £2.50). Dipstick tests had poorer cost-effectiveness than laboratory culture in children at intermediate risk of UTI (iNMB = £-1.41).

**Conclusions:**

Compared to GPs’ clinical judgement, high specificity clinical rules from the DUTY study could substantially reduce urine sampling, achieving lower costs and equivalent patient outcomes. Dipstick testing children for UTI is not cost-effective.

## Introduction

Urinary tract infection (UTI) is the fourth most common reason for prescribing antibiotics, accounting for approximately 8% of all antibacterial prescriptions [1]. Appropriate diagnosis and treatment of UTI in children presenting to primary care are particularly challenging because symptoms and signs are often nonspecific. The costs of a urine sample, laboratory test, and antibiotic are relatively low [2]. The economic impact may, however, be substantial because of the large number of acutely unwell children who present to primary care, additional diagnostic tests for structural abnormalities of the urinary tract [3], rare but serious complications of UTI, and the wider impact of antibiotic prescribing on bacterial resistance [4].

The few economic evaluations of UTI diagnosis in children [2], [5], [6] have compared the cost-effectiveness of urine tests once a urine sample has been obtained. There is very limited economic evidence to help primary care clinicians decide which children should have a urine sample taken and whether dipstick testing (DT) can guide therapy. Evidence is particularly needed for young children for whom current clinical guidelines of the National Institute for Health and Care Excellence [3] are not based on strong evidence of cost-effectiveness. In the Diagnosis of Urinary Tract infection in Young children (DUTY) study we report the development of a two-step clinical rule and demonstrate its superiority to routine clinical practice in the diagnosis of UTI in acutely unwell young children presenting to primary care in whom a clean catch urine sample was obtained. In this article we estimate the cost-effectiveness of these two steps. The first step evaluates whether clinical rules based on signs and symptoms identified in the DUTY study as predictive of UTI are more cost-effective than clinical judgment in identifying which children to test and treat for UTI. The second step evaluates the additional value of DT once a urine sample has been obtained.

## Methods

### The DUTY Study

The DUTY study was a multicenter, prospective, diagnostic cohort study that recruited children seeking care at National Health Service (NHS) primary care sites in Bristol, Cardiff, London, and Southampton. Children were eligible if younger than 5 years and with complaints of any acute (<28 days) illness episode that was associated with at least one potential marker for UTI [7]. Ethical approval was granted by the South West Southmead Research Ethics Committee (ref. #09/ H0102/64).

### Diagnostic Strategies

In the first step, we compared a “clinical judgment” diagnostic strategy with three strategies based on the DUTY “coefficient score” and four based on the simpler DUTY “points score.” Clinical judgment was defined by general practitioner (GP) responses to questions on the DUTY case report form about working diagnosis and planned management before urine sampling. In the clinical judgment strategy, the proportion of lower risk children was identified as those when the GP answered “No” to the question “If this child was NOT in the DUTY study would you have requested a urine sample?” or indicated a working diagnosis of “Not UTI.” Higher risk children were those when the GP had a working diagnosis of UTI and answered “Yes” to the question “Before seeing the dipstick results, are you planning on treating this child with antibiotics for suspected UTI?” Finally, intermediate-risk children were those when the GP had a working diagnosis of UTI and the GP answered “Yes” to the question “If this child was NOT in the DUTY study would you have requested a urine sample?”

The DUTY coefficient score is calculated from seven parent- or clinician-reported symptoms and signs, weighted according to the strength of independent association with UTI (see Appendix Table 1 in Supplemental Materials found at doi:10.1016/j.jval.2017.01.003). The simpler DUTY points score ranges from 0 to 9 and is calculated from a subset of five symptoms and signs that were dichotomized (e.g., present/absent) and assigned an integer score (i.e., 1 or 2) representing the strength of association with UTI (see Appendix Table 2 in Supplemental Materials found at doi:10.1016/j.jval.2017.01.003). The coefficient-based score is more accurate than the points-based score, but requires computational assistance to calculate. The cut points for the DUTY diagnostic strategies (see Appendix Table 3 in Supplemental Materials found at doi:10.1016/j.jval.2017.01.003) were selected to represent a range from more highly targeted (i.e., high specificity) to less highly targeted (i.e., high sensitivity) urine sampling strategies.

In the second step, we compared the short-term cost-effectiveness of immediate treatment on the basis of point-of-care DT versus delayed treatment after laboratory testing (LT). Nitrites and leukocytes (trace or more) were strongly and independently predictive of UTI in the DUTY study [7]. We evaluated two DT strategies: 1) immediate antibiotic if nitrite- *or* leukocyte-positive and 2) immediate antibiotic if nitrite- *and* leukocyte-positive.

### Model Overview

**Fig. 1:**
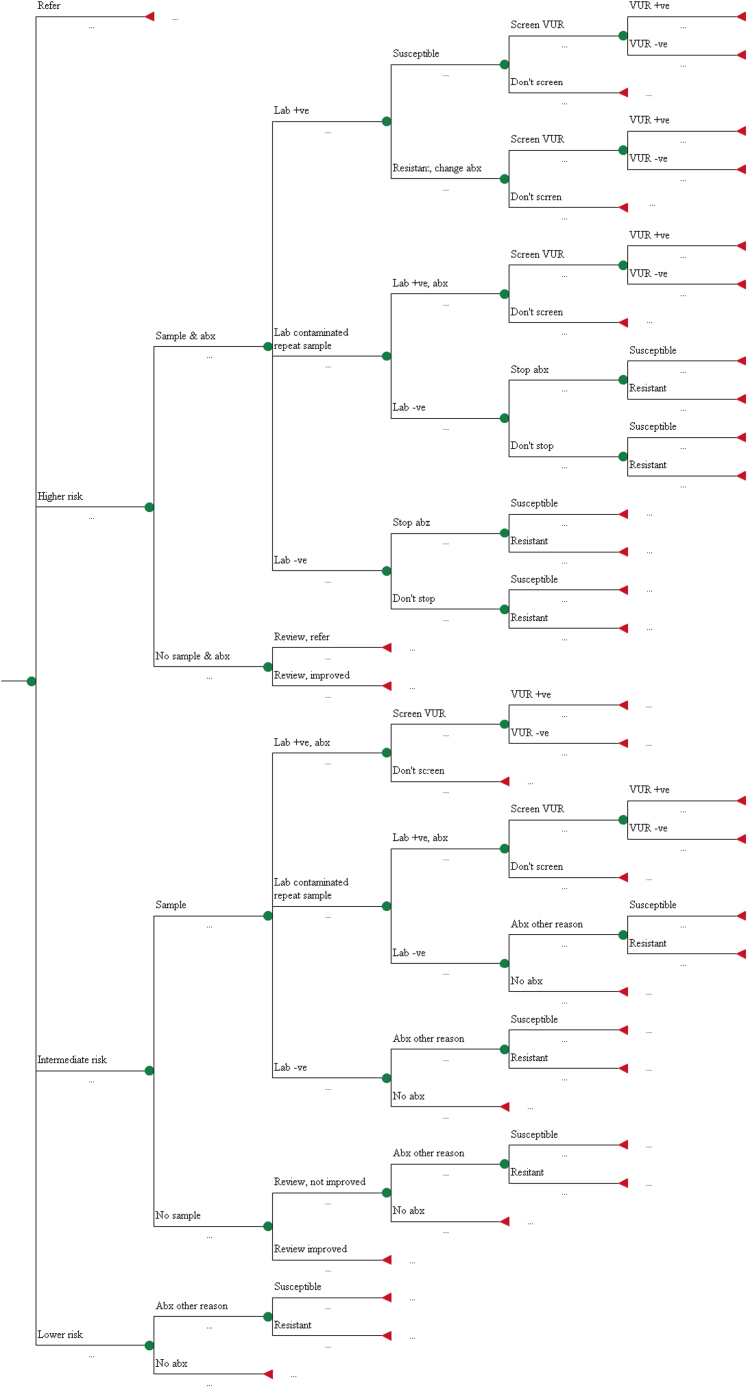
Decision tree for diagnosis and initial treatment of UTI

The overall model comprises several submodels. First, a short-term decision tree (Fig. 1) models testing and treatment during the index consultation. The acute illness phase is handled by a nine-state Markov model (see Appendix Figure 1 in Supplemental Materials found at doi:10.1016/j.jval.2017.01.003) estimating the time taken to recover (maximum 21 days) on the basis of the illness of the child and the treatment received. Correct UTI diagnosis can also lead to early diagnosis of vesicoureteral reflux (VUR), an abnormality that allows urine to flow backward from the bladder into the kidneys. Children with VUR are at increased risk of recurrent UTI (and subsequent pyelonephritic attacks [PAs]), although this can be reduced through prophylactic antibiotic treatment or surgery. Another Markov model (see Appendix Figure 2 in Supplemental Materials found at doi:10.1016/j.jval.2017.01.003) is used to calculate the number of recurrent UTIs and PAs in the 3 years after the index consultation. Finally, a long-term (lifetime) decision tree (see Appendix Figure 3 in Supplemental Materials found at doi:10.1016/j.jval.2017.01.003) models the impact of renal scarring in the earlier phases on the model, which is an important risk factor for long-term, potentially life-limiting renal complications such as end-stage renal disease. In each submodel, costs were estimated from a health service perspective and outcomes were expressed using quality-adjusted life-years (QALYs) or life-days (QALDs) [8].

Improved testing could lead to more targeted antibiotic treatment and quicker symptom resolution during the initial infection, better preventative treatment in the medium term leading to fewer renal scars (because of VUR treatment), and, consequently, fewer long-term complications.

### Short-Term Decision Tree

The structure of the decision tree (Fig. 1) is identical for all eight diagnostic strategies. Strategies differ in the proportion of children classified as lower, intermediate, or higher risk of UTI. We identified the proportion of children who are very unwell and referred directly to hospital for testing and treatment (for both UTI and non–UTI-related problems) as those when the GP answered “Yes” to the question “Before seeing the dipstick results, would you have referred this child to a pediatrician or admitted this child to hospital?” A urine sample is requested in children considered at higher risk of UTI and antibiotics prescribed. If laboratory culture demonstrates bacteriuria resistant to the prescribed antibiotic, the prescription will be changed. If no UTI is found, the GP may contact the parent to stop treatment. If the sample is contaminated, a repeat is sought. If no urine sample is obtained in a higher risk child, symptoms are reviewed in 2 days. If symptoms have not improved, the child is referred to hospital.

Urine sampling is attempted in children classified as intermediate risk of UTI, but antibiotic treatment is delayed until a positive laboratory result is returned. Children who cannot provide a sample are reviewed in 2 days and antibiotics are prescribed only if symptoms have not resolved and the working diagnosis is a (non-UTI) microbial infection. No urine sample is requested in children classified as lower risk of UTI, although antibiotics may be prescribed if the working diagnosis is a (non-UTI) microbial infection. Therefore, children in whom a UTI is undiagnosed may receive antibiotics serendipitously, although higher rates of uropathogen resistance to nontargeted antibiotics meant they often had slower symptom resolution than those with correctly identified UTIs. Children with UTI may be referred for ultrasound and, following a positive result, micturating cystourethrogram to test for VUR [3]. We assumed that children with a positive VUR diagnosis were treated with prophylactic antibiotics according to National Institute for Health and Care Excellence guidelines [3].

In the second step of our analysis we assumed that DT would be used to determine the initial treatment of children considered to be at intermediate risk of UTI on the basis of symptoms and signs (Fig. 1). Children with a positive dipstick result are prescribed antibiotics immediately, whereas no antibiotic is initiated until laboratory culture results are known in those with negative dipstick results.

### Acute Illness

Recovery in the 21 days after the initial consultation is modeled using a nine-state Markov process with a single-day cycle length (see Appendix Figure 1 in Supplemental Materials). Each health state has a cost and a utility score. The transition probabilities vary by state depending on the child’s health status (e.g., whether pyelonephritis is present) and treatment prescribed. For example, UTI promptly diagnosed and treated with an antibiotic will become asymptomatic more rapidly than undiagnosed and untreated UTI.

### Medium- and Long-Term Models

The medium-term model (see Appendix Figure 2 in Supplemental Materials) estimates the number of recurrent UTIs and PAs, with the associated costs and disutility, in the 3 years after the index consultation. We made the simplifying assumptions that children present with symptoms or signs potentially indicative of UTI at most annually and hence our cycle length was 1 year. Children with untreated VUR or previous UTI were at increased risk of recurrent UTI and PAs; these risks were, however, constant across all years of the medium-term model and were not affected by antibiotic treatment received during the index consultation. Costs and utilities of repeat presentations are identical to the index consultation and therefore dependent on the diagnostic strategy adopted. The long-term model (see Appendix Figure 3 in Supplemental Materials), which is based on previous work [2], calculates the lifetime cost, quality of life, and mortality consequences of the most severe UTI complications (progressive renal scarring [PRS] and end-stage renal disease [ESRD]). The probability of these complications increases with the number of PAs in the short- and medium-term phases of the model.

### Risk Stratification

Of the 3036 children in the DUTY providing a clean catch urine sample, we excluded those with a missing or contaminated research laboratory result (n = 346; 11.4%), dipstick result (n = 8; 0.3%), or information on GP clinical judgment (n = 6; 0.2%). Microbiologically confirmed UTI was defined as 10^5^ CFUs/ml (CFU, colony-forming unit) or more of a single or predominant uropathogen in the research laboratory culture. Contamination was defined as an NHS laboratory report of heavy mixed growth greater than 10^5^ CFUs/ml of more than two organisms [9].

In all strategies, 5% (133 of 2676) of children, including 5 with UTI, were reported by the GP to be “very unwell” and assumed to be referred to hospital for treatment. On the basis of clinical judgment, most of the remaining children (2276 of 2488; 91%) who did not have UTI were classified as lower risk, but only 56% (31 of 55) of children with UTI were classified as intermediate or higher risk (Table 1). When using the DUTY score, the DUTY5% and DUTY≥6 strategies had the highest specificity, whereas the DUTY20% and DUTY≥3 strategies had the highest sensitivity.

### Short-Term Model Parameters

In the DUTY study, 60 (2.2%) of 2676 children with an uncontaminated clean catch urine sample had a research laboratory–confirmed UTI (Table 2). Nine (16.3%) of 55 children with confirmed UTI and temperature recorded had fever (>38°C) and were assumed to have PA. The prevalence of VUR among children with UTI was estimated from a previous meta-analysis [10].

UTI resistance to non-UTI and UTI antibiotics was based on observed resistance (amoxicillin and trimethoprim, respectively) in the DUTY research laboratory reports. We estimated the proportion of children for whom an antibiotic was prescribed for another disease (not UTI) by calculating the proportion of children without UTI in DUTY whose parents reported antibiotic use within 2 days of the initial consultation. We assumed that 19% of children would return to primary care before symptom resolution [11], whereas the probability of further investigation of VUR was based on the proportion of DUTY children with UTI who had an ultrasound scan within 3 months. The estimates of the diagnostic accuracy of dipstick tests and NHS laboratory results were defined against the research laboratory in the DUTY study. Estimates of the diagnostic accuracy of ultrasound scans were taken from a previous meta-analysis [2].

### Symptom Resolution

Daily symptom resolution data were collected from parents in DUTY for 14 days after the consultation and used to estimate symptom resolution in children with treated UTI and in children without UTI (see Appendix Figure 4 in Supplemental Materials found at doi:10.1016/j.jval.2017.01.003). We extrapolated these results to 21 days using Weibull survival models because symptoms had not resolved by 14 days for some children. We estimated the symptom resolution rate in children with untreated UTI on the basis of a small randomized controlled trial comparing nitrofurantoin to placebo in women with bacteriologically proven UTI (Table 2) [12]. We assumed that symptom resolution was reduced by 30% when uropathogens were resistant to the prescribed antibiotic.

For children with delayed antibiotic treatment (e.g., waiting for a laboratory test result), we assumed that daily symptom resolution probabilities were the same as untreated UTI for the first 2 days. We assumed that the antibiotic treatment effect persisted for 7 days, meaning treated and untreated symptom resolution probabilities were identical between day 8 and day 21, and that all symptoms resolved by 21 days.

### Medium- and Long-Term Model Parameters

Estimates of the probability of primary care reconsultation with or without UTI, effectiveness of prophylactic treatment in children with VUR, long-term incidence of PRS and ESRD, and survival are presented in Table 2 and detailed elsewhere [9].

### Costs and Utilities

On the basis of observations in the DUTY study, the average time taken for a urine sample with and without a dipstick test was 12.0 minutes (cost £8.10) and 9.1 minutes (cost £7.03), respectively (see Appendix Table 4 in Supplemental Materials found at doi:10.1016/j.jval.2017.01.003). We assumed that GPs spent 45 seconds (cost £2.42) interpreting laboratory results and 5 minutes (cost £16.08) contacting parents to revise prescriptions. We estimated antibiotic costs on the basis of amoxicillin (125 mg/5 ml) for children treated for a non-UTI diagnosis and trimethoprim (50 mg/5 ml) for children with a UTI diagnosis. Other costs of initial care were based on a questionnaire completed by parents in the DUTY study 14 days postconsultation. We mapped drugs to British National Formulary codes [13] using the 2011 prescription cost analysis data set [14]. In the longer term we assumed that children with PRS have no increased costs of care until the onset of ESRD. Individuals with ESRD are treated by dialysis with an ongoing annual cost until death [15] or renal transplant with a treatment cost at the time of the procedure. All costs were inflated to 2014/2015 prices using the hospital and community health services pay and prices inflation factor [16].

In the absence of utility studies in infants with UTI [17], we used estimates from a study on rotavirus [18] (see Appendix Table 4 in Supplemental Materials) because the Preschool Children Quality Of Life (TAPQOL) questionnaire, administered to children in the DUTY study, demonstrated that health-related quality of life for children with UTI and gastroenteritis was similar (see Appendix Table 5 in Supplemental Materials found at doi:10.1016/j.jval.2017.01.003). We used utility values for pyelonephritis in adults reported in the literature [19]. We assumed that individuals with PRS experience no quality-of-life decrement until ESRD onset. Utility estimates for patients on dialysis and after renal transplant were estimated from previous research [20].

### Analysis

Data management was conducted in STATA (StataCorp, College Station, TX) and the model was implemented in Winbugs 1.4.3 (MRC biostatistics unit, Cambridge, UK) [21] using diffuse prior distributions for all parameters. The full model code is available in File 1 in Supplemental Materials found at http://dx.doi.org/10.1016/j.jval.2017.01.003. We calculated the expected costs, benefits, and incremental net monetary benefit (iNMB) [22] of each strategy compared with clinical judgment assuming the health service is willing to pay £20,000/QALY [23]. Outcomes and costs beyond the first year were discounted at 3.5% [23]. We assigned probability distributions to each model parameter and used Markov chain Monte-Carlo sampling to propagate parameter uncertainty through to the outcomes.

We undertook the following deterministic sensitivity analyses to evaluate the robustness of our conclusions to model assumptions: 1) increase in UTI prevalence to 10%; 2) perfectly accurate NHS laboratory cultures; 3) doubled antibiotic treatment effect; 4) doubled disutility from UTI infection; 5) simpler model excluding VUR and pyelonephritis; 6) doubled probability of PRS; and 7) doubled cost of ESRD.

## Results

### Diagnostic Accuracy and Treatment

The DUTY study GPs reported a working diagnosis of UTI in 9.1% of children when using clinical judgment including just over half of children who had a confirmed UTI (sensitivity = 56.4%; Table 3). Using the DUTY5% strategy, urine sampling could be approximately halved (4.8%) without any loss of sensitivity (58.2%). Alternatively, the DUTY10% strategy samples a similar proportion (9.6%) of children as clinical judgment, but has substantially higher sensitivity (70.9%). The most sensitive DUTY clinical rules (DUTY20% and DUTY≥3) achieved sensitivities in excess of 80%, but resulted in large increases in urine sampling (19.9% and 26.4%, respectively). The sensitivity of each strategy is reduced by the laboratory culture because NHS laboratories had imperfect diagnostic accuracy. Compared with clinical judgment, the DUTY10% rule results in a higher proportion of children with UTI treated with an antibiotic to which the bacterium was sensitive (56.4% vs. 49.2%). Nevertheless, a substantial proportion (>34%) of children with UTI receive either no or an inappropriate antibiotic under all strategies.

### Short-Term Costs and Outcomes

Mean sampling, laboratory culture, and antibiotic costs were the lowest in the high-specificity diagnostic strategies (e.g., DUTY≥6 £1.08; DUTY5% £1.22; and clinical judgment £1.99; Table 3). Short-term outcomes were very similar between diagnostic strategies. Short-term average QALDs were 20.73 for all strategies, whereas the number of asymptotic days ranged from 16.34 to 16.35, although small differences existed at the third and fourth decimal places. These similarities are driven by the low prevalence of UTI, the small differences in diagnostic accuracy of strategies, and the limited effect of antibiotics on acute symptom duration.

The high-specificity DUTY clinical rules (DUTY5%, DUTY≥6, and DUTY≥5) were more cost-effective than clinical judgment in the short-term (iNMB = £0.78, £0.84, and £0.42, respectively; Table 3). These efficiencies are predominantly due to financial savings arising from fewer, better targeted, urine samples compared with clinical judgment. The DUTY10% rule had similar short-term cost-effectiveness to clinical judgment (iNMB = £0.00). For the highest sensitivity DUTY clinical rules (DUTY20%, DUTY≥4, and DUTY≥3), the benefit of identifying and treating slightly more UTIs was outweighed by the higher costs of sampling and testing substantial numbers of children (iNMB = −£1.69, −£1.93, −£2.61, respectively).

### Medium- and Long-Term Costs and Outcomes

In the medium and long-term, diagnostic strategies with the highest sensitivity led to VUR treatment in a larger proportion of cases and had slightly lower rates of UTI recurrence (Table 4). Nevertheless, differences between strategies in life expectancy and QALYs were negligible. The high-specificity diagnostic strategies (i.e., DUTY5%, DUTY≥6, and DUTY≥5) were more cost-effective than clinical judgment in the long-term (iNMBs £2.31, £2.50, and £1.22, respectively). Even small differences in net benefits per child are important given the large number of children with acute illness presenting to primary care (Table 4, final row).

### Laboratory versus Dipstick-Based Treatment

In the DUTY5% strategy, 2.3% of children (18.8% of whom have UTI) are considered intermediate risk (Table 1) and, in the absence of a dipstick test, clinicians would have delayed treatment pending the laboratory test result. DT and treatment for children with positive leukocytes and nitrites result in 25.0% of those with intermediate risk of UTI receiving immediate antibiotics to which the bacterium was sensitive (Table 5). Nevertheless, DT increased the proportion of children without UTI incorrectly treated compared with LT (3.3% vs. 2.3%). Average sampling, testing, and treatment costs are higher in this DT strategy (£17.13) than in the LT strategy (£15.66), mainly because of the additional time and cost of the dipstick test. Both DT strategies had poorer cost-effectiveness compared with those based on LT of children at intermediate risk of UTI (iNMB = −£1.41 for leukocytes and nitrites; iNMB = −£1.91 for leukocytes or nitrites).

### Sensitivity Analysis

Both the probabilistic (Table 3, Table 4) and deterministic sensitivity analyses (see Appendix Tables 6 and 7 in Supplemental Materials found at doi:10.1016/j.jval.2017.01.003) indicate that the finding that the DUTY5% strategy is more cost-effective than clinical judgment is robust to substantial changes in key model parameters. Our short-term results were similar when using a simplified model that did not seek to estimate the impact of VUR or pyelonephritis, suggesting that these elements did not play an important role in driving our conclusions.

## Discussion

### Summary of Findings

We evaluated the cost-effectiveness of a two-step clinical rule using symptoms, signs, and dipstick test results to select children for urine sampling and antibiotic treatment. Compared with GPs’ clinical judgment, the DUTY5% clinical rule could substantially reduce urine sampling, achieving lower costs and equivalent patient outcomes. DUTY points-based rules are more cost-effective than clinical judgment at high-specificity thresholds (DUTY≥5 and DUTY≥6) and could be used when it is infeasible to estimate the DUTY coefficient-based score. Our findings suggest that urine sampling should be more carefully targeted, rather than increased, but do not support the use of DT in children at intermediate risk of UTI. The benefits of immediate dipstick-guided treatment were counterbalanced by imperfect test specificity resulting in more antibiotic prescriptions in children without UTI.

### Strengths and Limitations

Our model was based on individual patient data from a large, rigorously conducted, prospective diagnostic cohort study. Therefore, most of the parameters underlying the short-term model come from a consistent, high-quality data source. In the DUTY study, urine samples were analyzed by both health service and research laboratories providing more accurate estimates of the prevalence of UTI and contamination. Furthermore, we were able to model the impact of false-negative laboratory results and antibiotic resistance on the efficiency of UTI diagnosis. Our results are based on evidence from children for whom clean catch samples were collected and are not necessarily generalizable to younger children for whom nappy pads are generally used for sampling. The clinical judgment diagnostic strategy aimed to represent current practice, on the basis of clinicians’ responses to questions about working diagnoses and testing and treatment plans. DUTY study participation may have, however, sensitized clinicians to the possibility of UTI, leading to an overestimate of urine sampling rates. Although this would not alter our conclusion that selected symptoms and signs can help primary care clinicians to target urine sampling, it does strengthen the interpretation that high-specificity diagnostic strategies (e.g., DUTY5% and DUTY≥5) are most likely to be cost-effective in diagnosing and treating UTI.

Some of the evidence underlying the model was imprecise and potentially biased. For example, there is no randomized controlled trial–based evidence on the effect of antibiotics in young children with UTI. The evidence underlying the long-term model is based on observational associations between recurrent UTI and renal disease, which continues to evolve: the Randomized Intervention for Children with Vesicoureteral Reflux (RIVUR) trial comparing daily trimethoprim-sulfamethoxazole prophylaxis to placebo in children with VUR recently reported a 50% reduction in recurrent UTI, but no trend for reduced incidence of renal scarring [24]. Similarly, the choice of sampling distribution for some parameters, in particular costs and utilities, was based on convention rather than on primary data, introducing subjectivity into the probabilistic sensitivity analysis.

Our model did not include other potential long-term consequences of UTI such as pregnancy-related complications or hypertension in which the causal role of UTI is debated and difficult to ascertain [25], [26]. It is possible that other long-term consequences of UTI exist. Identifying and including these would favor more sensitive strategies. Nevertheless, our conclusions were insensitive to different assumptions about the long-term sequelae of UTI. The model results are dominated by the short-term costs of testing and treating rather than by the long-term sequelae because most children presenting to primary care do not have UTI, most children with UTI will not develop ESRD, and each strategy has only a small impact on the proportion of children treated appropriately.

The large number of risk thresholds and the multiple ways of using DT and laboratory culture to guide treatment produce an almost unlimited number of potential management strategies. We evaluated some that closely reflect current practice, but other unevaluated strategies could prove more cost-effective. We did not quantify the societal costs of antibiotic resistance. Current methods may underestimate the cost of antibiotic resistance, and accurate estimation may not be possible [4]. Given increasing levels of resistance and the paucity of new antibiotics, the inclusion of these costs would further strengthen the case for high-specificity diagnostic strategies that limit prescriptions to those most likely to have a UTI.

### Results in Context with Other Studies

As far as we are aware, this is the first study to evaluate the cost-effectiveness of a clinical rule to identify children with UTI in primary care. Previous work has assessed the most cost-effective test or series of tests for diagnosing UTI rather than evaluating which children should be considered at risk of UTI. An economic model evaluating testing strategies for children with suspected UTI concluded that either presumptive treatment or treatment based on positive dipstick nitrites and leukocytes and micturating cystourethrogram was optimal [2]. Our findings suggest that waiting for a positive laboratory culture is more cost-effective in children at intermediate risk of UTI. The differences in findings are likely to be partly due to the inclusion of serendipitous treatment and detailed daily symptom resolution rates in our model.

### Clinical and Research Implications

Each year large numbers of young children present to primary care with acute illness. Therefore, even small modifications to diagnostic strategies for common conditions such as UTI will have a large impact on aggregate costs and workload. Our findings demonstrate the need for clinicians to base the decision to collect a urine sample on symptoms and signs known to be predictive of UTI in primary care rather than on personal judgment or on evidence derived from secondary care. Our results also illustrate the trade-off between the small but certain short-term costs of UTI diagnosis and treatment against the important but less certain benefits of detecting and treating UTI and potentially preventing renal disease.

Our findings suggest that clinicians should select low-cost, high-specificity diagnostic strategies. A GP requesting urine samples in children by using the DUTY5% strategy would sample 4.8% of all acutely unwell children and request a sample in 58% of children who have UTI at a testing and antibiotic cost of £1.22 per child. In settings where symptoms and signs are routinely recorded in electronic records, this process could be automated. Nevertheless, in settings where resources do not permit this, a GP using the DUTY≥5 strategy would sample 6.7% of all acutely unwell children, including 53% of children who have UTI, at a testing and antibiotic cost of £1.57 per child. Both strategies are more cost-effective than clinical judgment alone.

Our research does not support the routine use of DT to guide treatment. This conclusion is, however, based on weak evidence about the effect of antibiotics. Trial evidence comparing the cost-effectiveness of management strategies in women with suspected UTI concluded that all strategies achieved similar symptom control and that dipstick test–guided management was likely, albeit with considerable uncertainty, to be cost-effective [27], [28]. A similar trial of management strategies in infants is needed. Studies of parent-reported quality of life and disutility of UTI symptoms in young children would enable more precise estimates of short-term benefits of antibiotics. Long-term epidemiological study designs are needed to better quantify and understand the association between childhood UTI and renal disease.

## Conclusions

The DUTY study coefficient and point scores were more cost-effective than GPs’ clinical judgment in selecting children for urine sampling and treatment for UTI. Small differences between strategies in cost-effectiveness are important given the large number of urine samples collected in children. High-specificity thresholds, such as DUTY≥5, are simple to implement and likely to be most cost-effective than clinical judgment. Our findings do not support the routine use of DT, but trial evidence is needed to compare the cost-effectiveness of various management strategies.

## Figures and Tables

**Table 1:** Risk stratification for clinical judgment and DUTY clinical rules*

**Diagnostic strategy**	**Actual UTI status**	**Risk strata**			
		**Lower**	**Intermediate**	**Higher**	**Multinomial distribution**
Clinical judgment	No UTI (%)	2276 (91.48)	83 (3.34)	129 (5.18)	(2276, 83, 129)
	UTI (%)	24 (43.64)	9 (16.36)	22 (40.00)	(24, 9, 22)
DUTY5%	No UTI (%)	2393 (96.18)	52 (2.09)	43 (1.73)	(2393, 52, 43)
	UTI (%)	23 (41.82)	12 (21.82)	20 (36.36)	(23, 12, 20)
DUTY10%	No UTI (%)	2271 (91.28)	122 (4.90)	95 (3.82)	(2271, 122, 95)
	UTI (%)	16 (29.09)	7 (12.73)	32 (58.18)	(16, 7, 32)
DUTY20%	No UTI (%)	2004 (80.55)	267 (10.73)	217 (8.72)	(2004, 267, 217)
	UTI (%)	8 (14.55)	8 (14.55)	39 (70.91)	(8, 8, 39)
DUTY≥6	No UTI (%)	2395 (96.26)	82 (3.30)	11 (0.44)	(2395, 82, 11)
	UTI (%)	31 (56.36)	17 (30.91)	7 (12.73)	(31, 17, 7)
DUTY≥5	No UTI (%)	2340 (94.05)	55 (2.21)	93 (3.74)	(2340, 55, 93)
	UTI (%)	26 (47.27)	5 (9.09)	24 (43.64)	(26, 5, 24)
DUTY≥4	No UTI (%)	1946 (78.22)	394 (15.84)	148 (5.95)	(1946, 394, 148)
	UTI (%)	11 (20.00)	15 (27.27)	29 (52.73)	(11, 15, 29)
DUTY≥3	No UTI (%)	1829 (73.51)	511 (20.54)	148 (5.95)	(1829, 511, 148)
	UTI (%)	8 (14.55)	18 (32.73)	29 (52.73)	(8, 18, 29)

DUTY, Diagnosis of Urinary Tract infection in Young children; UTI, urinary tract infection.

⁎ Excludes patients who did not have UTI results, those referred immediately to secondary care, or when missing data did not allow calculation of clinical judgment decision.

**Table 2:** Parameters used to estimate diagnosis and treatment pathways and health status

**Parameter**	**Estimate**	**Distribution**	**Source**
*Short-term model*			
UTI prevalence	0.022	Binomial (60, 2676)	DUTY
PA (among those with UTI)	0.164	Binomial (9, 55)	DUTY
VUR (among those with UTI)	0.240	Odds~LN* (−1.153, 0.113)	[10]
Very unwell†	0.050	Binomial (133, 2676)	DUTY
Urine sample obtained‡	0.957	Binomial (2231, 2332)	DUTY
Contamination	0.046	Binomial (140, 2619)	DUTY
Antibiotic resistance (amoxicillin)	0.531	Binomial (50, 94)	DUTY
Antibiotic resistance (trimethoprim)	0.277	Binomial (26, 94)	DUTY
Reconsultation	0.189	Binomial (42, 222)	[11]
Antibiotics for non-UTI reason	0.294	Binomial (78, 262)	DUTY
Stop antibiotic given no UTI	0.075	Uniform (0.05, 0.10)	Expert opinion
Referred for US	0.059	Binomial (6, 103)	DUTY
Dipstick (L or N)			DUTY
Sensitivity	0.767	Binomial (46, 60)	
Specificity	0.841	Binomial (2200, 2616)	DUTY
Dipstick (L and N)			DUTY
Sensitivity	0.367	Binomial (22, 60)	
Specificity	0.989	Binomial (2588, 2616)	DUTY
Laboratory test§			DUTY
Sensitivity	0.789	Binomial (45, 57)	
Specificity	0.976	Binomial (2341, 2398)	DUTY
US for VUR			[2]
Sensitivity	0.440	Odds~LN (−0.243, 0.2352)	
Specificity	0.775	Odds~LN (1.238, 0.2862)	[2]
MCUG for VUR	1.000	Fixed	Assumption
Sensitivity			
Specificity	1.000	Fixed	Assumption
Antibiotic treatment effect||	0.550	RR~LN (−0.599, 0.247)	[12]
Reduced effect in resistant bacteria	0.700	Uniform (0.5, 0.9)	Expert opinion
*Medium-term model*			
Consult—no UTI	0.693	Binomial (21193, 30588)	[29]
Consult—UTI, no history¶	0.003	Binomial (9.33, 2789)	[30]
Consult—UTI, history, and no/treated VUR	0.080	Odds~LN (−2.442, 0.2182)	[10]
Treatment effect for treated VUR#	0.68	RR~LN (−0.385, 0.2802)	[31]
*Long-term model*			
PRS			[25]
0 PA	0.050	Binomial (7, 141)	
1 PA	0.087	Binomial (32, 366)	
2 PA	0.161	Binomial (15, 93)	
3 PA	0.343	Binomial (12, 35)	
4 PA	0.583	Binomial (14, 24)	
ESRD given PRS	0.050		[32]
Mean age of ESRD onset	13.67	Triangle (7, 24)	[33], [34]
Transplant	0.500		Assumption
Dialysis	0.500		Assumption
Years survival—no ESRD	73.00	Uniform (69.4, 76.7)	[35]
Years survival—dialysis	12.25	Uniform (11.6, 12.9)	[36]
Years survival—transplant	21.60	Uniform (20.5, 22.7)	[37]

ESRD, end-stage renal disease; GP, general practitioner; MCUG, micturating cystourethrogram; L, leukocytes; N, nitrates; NHS, National Health Service; PA, pyelonephritic attack; PRS, progressive renal scarring; US, ultrasound; UTI, urinary tract prevalence; VUR, vesicoureteral reflux.

⁎ Lognormal.

† GP answered “Yes” to the question “Before seeing the dipstick results, would you have referred this child to a pediatrician or admitted this child to hospital.”

‡ On the basis of the proportion of children older than 3 y for whom a sample was obtained.

§ After removing samples that were found to be contaminated in the NHS laboratory.

|| Risk ratio comparing symptom resolution rates for children not treated with antibiotics to those in children treated with antibiotics.

¶ Numerator adjusted to account for 18-mo follow-up period.

# Relative risk comparing UTI recurrence rates in children with VUR with children without VUR.

**Table 3:** Short-term costs and benefits of seven DUTY diagnostic strategies compared with clinical judgment

**Costs and outcomes**	**Clinical judgment**	**DUTY5%**	**DUTY10%**	**DUTY20%**	**DUTY≥6**	**DUTY≥5**	**DUTY≥4**	**DUTY≥3**
Diagnostic pathway								
Urine sample requested (%)	9.12	4.79	9.61	19.89	4.40	6.65	21.94	26.43
Sensitivity—urine sampling	0.564	0.582	0.709	0.854	0.436	0.527	0.800	0.854
Specificity—urine sampling	0.915	0.962	0.913	0.805	0.963	0.941	0.782	0.735
Sensitivity—after laboratory test*	0.426	0.439	0.536	0.645	0.330	0.398	0.604	0.645
Specificity—after laboratory test†	0.998	0.999	0.998	0.996	0.999	0.999	0.995	0.994
Treatment pathway (children with UTI)								
Immediate, appropriate‡ antibiotic (%)	36.64	34.05	46.48	52.80	20.67	39.48	41.75	41.03
Laboratory informed§, appropriate antibiotic (%)	12.51	16.55	9.94	11.40	23.14	7.12	20.74	24.81
Inappropriate antibiotic (%)	17.56	16.50	20.01	21.22	12.79	18.83	17.68	17.03
No antibiotic (%)	33.29	32.90	23.56	14.59	43.40	34.58	19.83	17.14
Treatment pathway (children without UTI)								
Antibiotic treatment for UTI (%)	4.79	1.62	3.56	8.16	0.47	3.45	5.75	5.85
Short-term costs and outcomes								
*Costs per child*								
Sampling, culture, antibiotic treatment costs	1.99	1.22	2.05	3.80	1.08	1.57	3.99	4.68
Initial (21 d) health service costs	44.06	43.28	44.07	45.78	43.19	43.63	46.01	46.69
Outcomes								
Asymptomatic days	16.34	16.34	16.35	16.35	16.34	16.34	16.35	16.35
Short-term average QALDs	20.73	20.73	20.73	20.73	20.73	20.73	20.73	20.73
Cost-effectiveness								
iNMB|| per child (95% CI)	–	0.78 (0.76 to 0.79)	0.00 (0.00 to 0.01)	−1.69 (−1.71 to 1.68)	0.84 (0.83 to 0.85)	0.42 (0.41 to 0.43)	−1.93 (−1.95 to 1.92)	−2.61 (−2.63 to −2.59)

CI, confidence interval; DUTY, Diagnosis of Urinary Tract infection in Young children; iNMB, incremental net monetary benefit; QALDs, quality-adjusted life-days; UTI, urinary tract infection.

⁎ The proportion of children with UTI whose urine is sampled and the laboratory culture is positive.

† The proportion of children without UTI whose urine is not sampled or the laboratory culture is negative.

‡ (In)appropriate defined as an antibiotic to which the bacterium is (not) sensitive.

§ Antibiotic prescribing determined by laboratory result, usually started a few days after primary care attendance.

|| On the basis of a £20,000/QALY threshold; compared with clinical judgment strategy (bootstrapped 95th percentile CI); a positive value indicates that the strategy is more cost-effective than clinical judgment.

**Table 4:** Medium- and long-term costs and benefits of seven DUTY diagnostic strategies compared with clinical judgment

**Costs and outcomes**	**Clinical judgment**	**DUTY5%**	**DUTY10%**	**DUTY20%**	**DUTY≥6**	**DUTY≥5**	**DUTY≥4**	**DUTY≥3**
Average number of UTI recurrence at 3 y/10,000 patients	165.5	165.5	165.5	165.4	165.5	165.5	165.5	165.4
% ESRD*	0.250	0.250	0.250	0.250	0.250	0.250	0.250	0.250
Average years lived	72.94	72.94	72.94	72.94	72.94	72.94	72.94	72.94
Average lifetime cost	182.3	179.9	182.2	187.3	179.7	181.0	188.1	190.1
Average lifetime QALY	25.74	25.74	25.74	25.74	25.74	25.74	25.74	25.74
iNMB†, per child (95% CI)	–	2.31 (2.30 to 2.33)	0.00 (−0.01 to 0.01)	−5.00 (−5.03 to −4.97)	2.50 (2.49 to 2.51)	1.22 (1.21 to 1.23)	−5.78 (−5.81 to −5.75)	−7.78 (−7.82 to −7.74)
iNMB, annual UK‡	–	£10.75M	£0.00M	−£23.25M	£11.63M	£5.67M	−£25.88M	−£36.18M

CI, confidence interval; ESRD, end-stage renal disease; iNMB, incremental net monetary benefit; QALY, quality-adjusted life-year; UTI: urinary tract infection.

⁎ Strategies are estimated to have no impact on lifetime ESRD or QALYs to three decimal places because most children do not have UTI, most children with UTI will not develop ESRD, each strategy has only a small impact on the proportion of children with UTI treated inappropriately, and the effect of prophylaxis on ESRD incidence is uncertain.

† On the basis of a £20,000/QALY threshold; compared with clinical judgment strategy (bootstrapped 95th percentile CI); a positive value indicates that the strategy is more cost-effective than clinical judgment.

‡ Assuming one consultation per annum with acute illness when a clean catch sample could be collected for each of UK’s 4.65 million children younger than 5 y.

**Table 5:** Costs and benefits of DT compared with LT in children judged to be intermediate risk for UTI

**Costs and outcomes**	**DUTY5%**		
	**LT**	**DT (L or N)**	**DT (L and N)**
Diagnostic pathway			
Dipstick test (%)	0.00	95.67	95.67
Treatment pathway (children with UTI)			
Immediate, appropriate antibiotic (%)	0.00	52.22	24.98
Treatment determined by laboratory culture, appropriate antibiotic (%)	78.45	18.39	49.73
Inappropriate antibiotic (%)	3.32	22.00	12.25
No antibiotic (%)	18.23	7.39	13.05
Treatment pathway (children without UTI)			
Antibiotic treatment for UTI (%)	2.27	17.13	3.27
Short-term costs and outcomes			
*Costs per patient*			
Sampling, dipstick, culture, and antibiotic treatment	15.66	17.70	17.13
Total short-term cost	57.71	59.66	59.14
Outcomes			
Asymptomatic days	16.34	16.35	16.35
Short-term average QALDs	20.73	20.73	20.73
Summary measure			
iNMB, per child (95% CI)*	–	−1.91 (−1.99 to −1.83)	−1.41 (−1.50 to −1.32)

CI, confidence interval; DT, dipstick-based treatment; DUTY, Diagnosis of Urinary Tract infection in Young children; iNMB, incremental net monetary benefit; L, leukocytes; LT, laboratory-based treatment; N, nitrates; QALDs, quality-adjusted life-days; QALY, quality-adjusted life-year; UTI, urinary tract infection.

⁎ On the basis of a £20,000/QALY threshold; compared with clinical judgment strategy (bootstrapped 95th percentile CI); a positive value indicates that the strategy is more cost-effective than clinical judgment.

## Footnotes

Supplemental material accompanying this article can be found in the online version as a hyperlink at doi:10.1016/j.jval.2017.01.003 or, if a hard copy of article, at www.valueinhealthjournal.com/issues (select volume, issue, and article).
