# Supplementary material for: The Diagnosis of Urinary Tract Infection in Young Children (DUTY) Study Clinical Rule: Economic Evaluation
Source: Value Health. 2017 Apr;20(4):556–66. doi: 10.1016/j.jval.2017.01.003 (PMC5406157; doi:10.1016/j.jval.2017.01.003)
Supplement: Supplementary file 1 — Supplementary material [file mmc1.docx]

Supplemental table 1: DUTY coefficient elements and weights^[[1]](#footnote-1)^

| **Index tests** | **Odds Ratio (95% CI)** |
| --- | --- |
| **Pain/crying when passing urine** | |
| No problem | 1 (ref) |
| Slight problem | 1.73 (0.73-4.06) |
| Moderate problem | 4.80 (2.30-10.04) |
| Severe problem | 15.81 (7.37-33.89) |
| **Smelly urine** | |
| No problem | 1 (ref) |
| Slight problem | 4.28 (2.02-9.05) |
| Moderate problem | 5.14 (2.60-10.19) |
| Severe problem | 8.76 (3.76-20.41) |
| **Previous UTI** | |
| No | 1 (ref) |
| Yes | 2.66 (1.34-5.26) |
| **Cough** |  |
| No problem | 1 (ref) |
| Slight problem | 1.32 (0.68-2.55) |
| Moderate problem | 1.38 (0.72-2.68) |
| Severe problem | 0.29 (0.09-0.97) |
| **Clinician global impression of illness severity (0-10)** | |
| 0-1 | 1 (ref) |
| 2 | 1.98 (0.93-4.19) |
| 3 | 2.72 (1.28-5.81) |
| 4-5 | 3.87 (1.72-8.73) |
| 6 or more | 7.24 (2.59-20.25) |
| **Abdominal examination: any tenderness** | |
| No | 1 (ref) |
| Yes | 2.24 (0.95-5.25) |
| **Ear examination: any acute abnormality** | |
| No | 1 (ref) |
| Yes | 0.27 (0.10-0.74) |

Supplemental table 2: DUTY points elements and weights^[[2]](#footnote-2)^

| **Clinical Characteristic (Present / Absent)** | **Points** |
| --- | --- |
| Pain/crying passing urine | 2 |
| Smelly urine | 2 |
| Previous UTI | 1 |
| Absence of severe cough | 2 |
| Severe illness present | 2 |

Supplemental table 3: Overview of the DUTY algorithm

| **Strategy** | **Intermediate risk cut-point** | **% of children above intermediate cut-point** | **Higher risk cut-point** | **% of children above higher cut-point** |
| --- | --- | --- | --- | --- |
| **Coefficient-based algorithm^[[3]](#footnote-3)^** |  |  |  |  |
| DUTY5% | 0.083 | 4.99 | 0.142 | 2.48 |
| DUTY10% | 0.041 | 10.07 | 0.083 | 4.99 |
| DUTY20% | 0.020 | 20.88 | 0.041 | 10.07 |
| **Points-based algorithm^[[4]](#footnote-4)^** |  |  |  |  |
| DUTY≥6 | 6 | 4.60 | 7 | 0.71 |
| DUTY≥5 | 5 | 6.96 | 6 | 4.60 |
| DUTY≥4 | 4 | 23.04 | 5 | 6.96 |
| DUTY≥3 | 3 | 27.76 | 5 | 6.96 |

Supplemental figure 1: Short-term Markov model^[[5]](#footnote-5)^ ^[[6]](#footnote-6)^


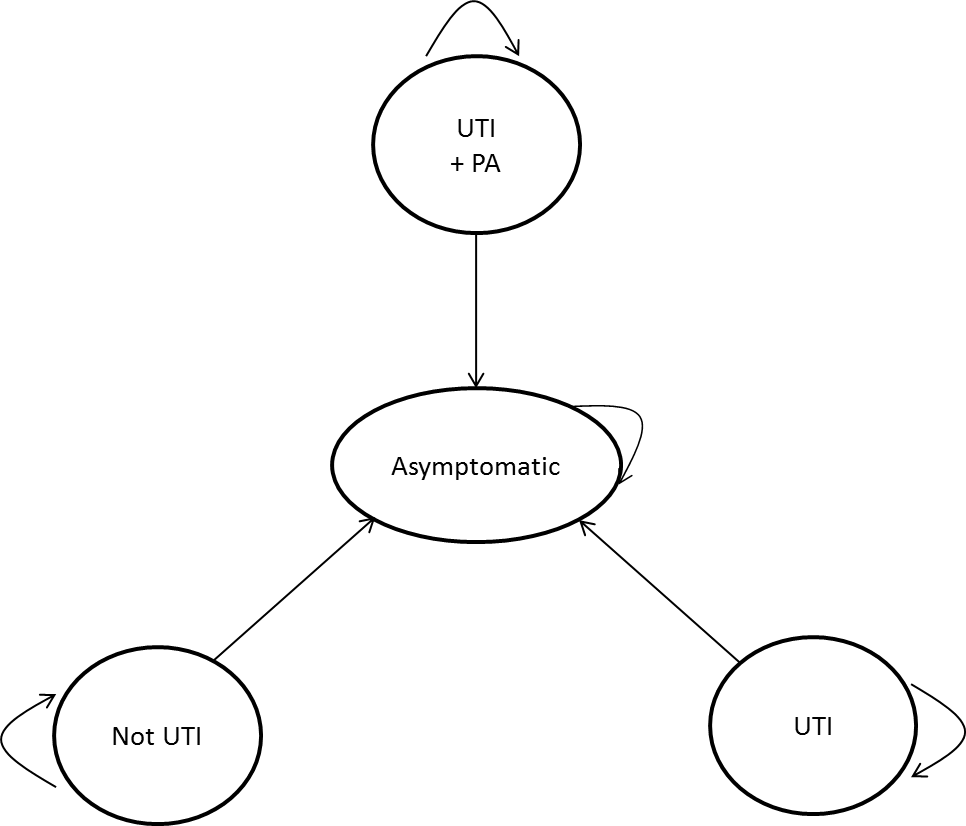


Supplemental figure 2a: Medium-term model*^[[7]](#footnote-7)^*


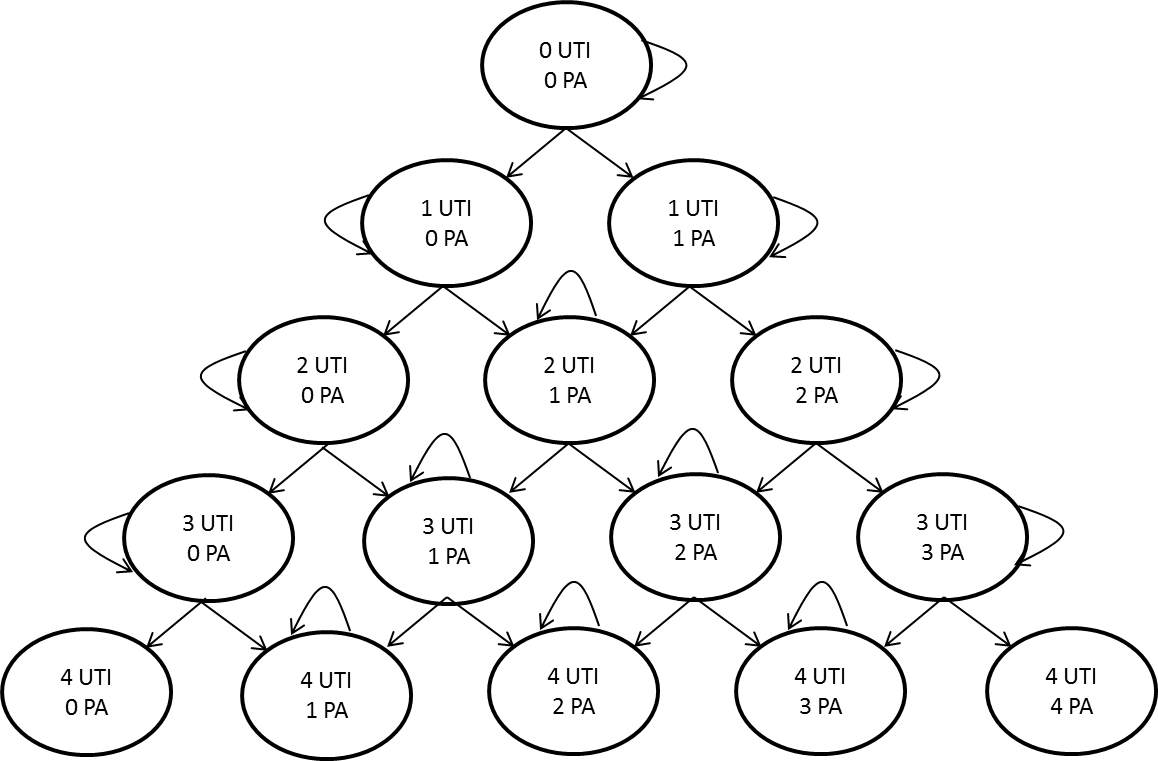


Supplemental figure 2b: Medium-term model, no VUR.^[[8]](#footnote-8)^

**
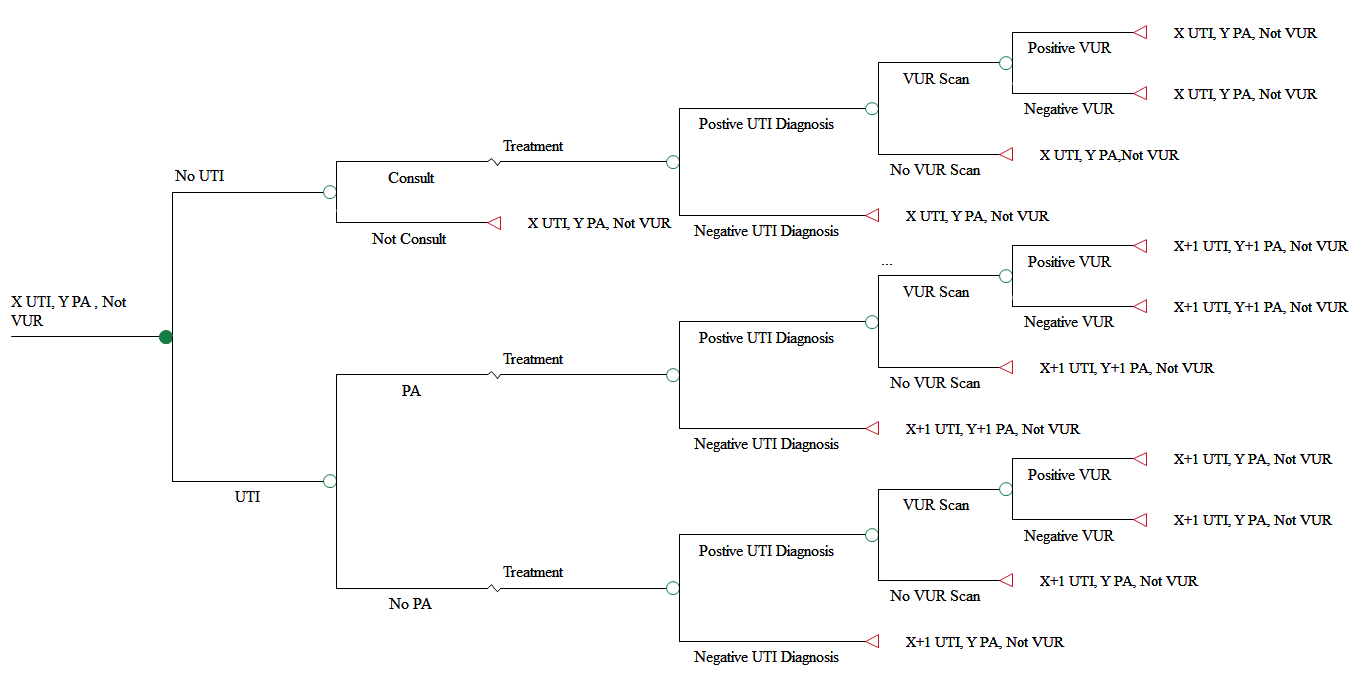
**

Supplemental figure 2c: Medium-term model, treated VUR.


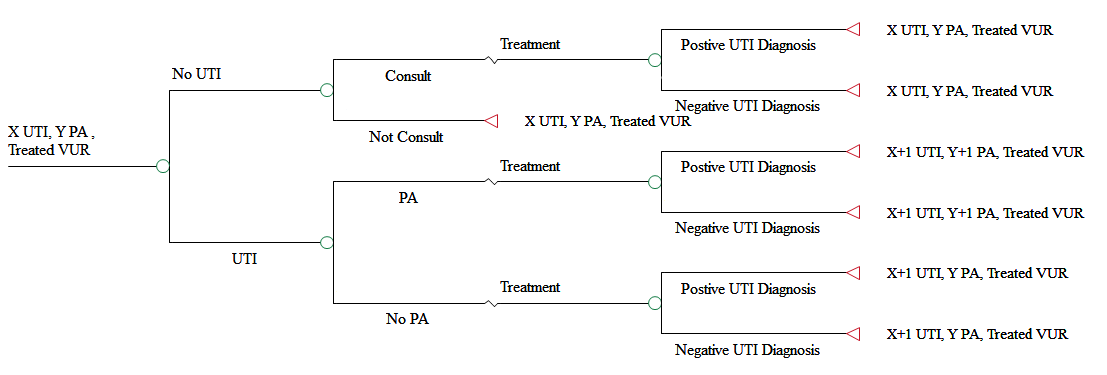


Supplemental figure 2d: Medium-term model, untreated VUR.

**
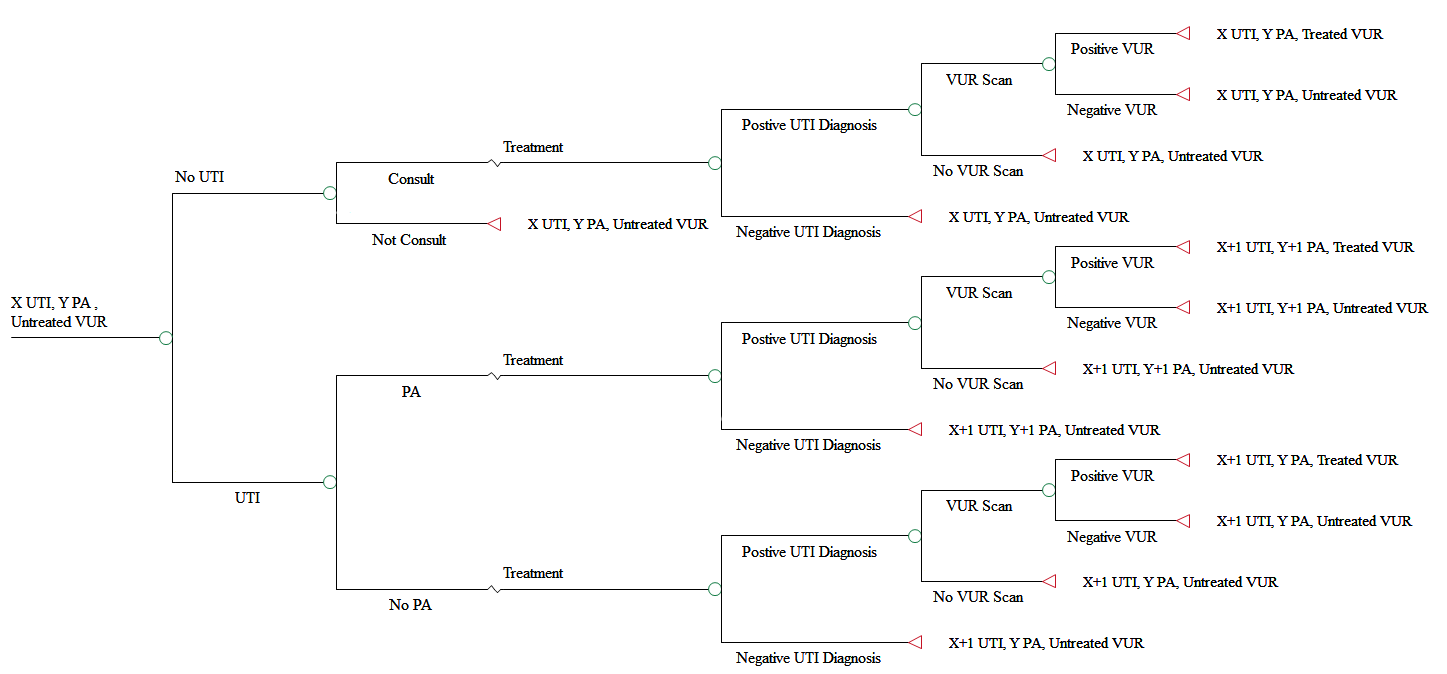
**

Supplemental figure 3: Long-term model^[[9]](#footnote-9)^

**
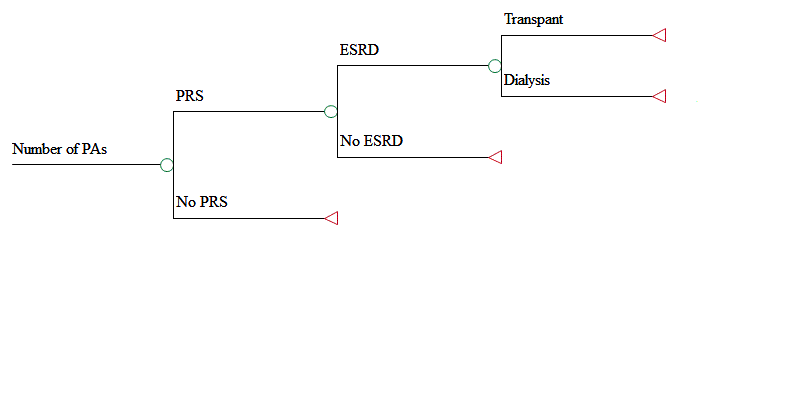
**

Supplemental figure 4: Comparison of observed and modelled^[[10]](#footnote-10)^ symptom resolution for children a) with treated urinary tract infection (n=23) and b) without urinary tract infection (n=291)

| a)   |
| --- |
| b)   |

Supplemental table 4: Cost and quality of life (utility) parameters^[[11]](#footnote-11)^

| **Item** | **Estimate (£)^[[12]](#footnote-12)^** | **Distribution** | **Source** |
| --- | --- | --- | --- |
| Urine sample | 7.03 | Uniform(3.52,10.55) | DUTY |
| Urine sample & dipstick | 8.10 | Uniform(4.05,12.15) | DUTY |
| Sample attempted but not obtained | 1.46 | Uniform(0.73,2.19) | DUTY |
| Laboratory test | 6.36 | Uniform(3.18,9.54) | Carter Report^1^ |
| Dipstick test kit | 0.39 | Uniform(0.2,0.59) | DUTY |
| GP test interpretation | 2.42 | Uniform(1.21,3.63) | Expert Opinion |
| GP call to parents | 16.08 | Uniform(8.04,24.12) | Expert Opinion |
| Trimethoprim | 2.91 | Uniform(1.45,4.36) | PCA^2^ |
| Amoxicillin | 1.29 | Uniform(0.64,1.93) | PCA^2^ |
| Ultrasound | 51.88 | Uniform(25.94,77.81) | NRC^3^ |
| MCUG | 142.20 | Uniform(71.1,213.3) | Whiting^4^ |
| UTI cost (2 days) | 33.73 | Uniform(16.86,50.59) | DUTY |
| UTI daily cost | 11.49 | Uniform(5.74,17.23) | DUTY |
| UTI & PA cost (2 days) | 50.60 | Uniform(15.92,47.76) | DUTY & Expert Opinion |
| UTI & PA daily cost | 17.23 | Uniform(8.62,25.85) | DUTY & Expert Opinion |
| Non UTI cost (2 days) | 24.68 | Uniform(12.34,37.02) | DUTY |
| Non UTI daily cost | 5.49 | Uniform(2.74,8.23) | DUTY |
| Antibiotic Prophylaxis | 24.69 | Uniform(12.35,37.04) | Nagler^5^, PCA^2^ |
| Dialysis per year | 22,467 | Uniform(11233.53,33700.59) | Baboolal^6^ |
| Transplant^[[13]](#footnote-13)^ | 20,186 | Uniform(10093,30278) | NRC^3^ |
| **Utilities** |  |  |  |
| Well Child | 1.000 | Uniform(0.80,1.00) | Assumption |
| UTI/ No PA | 0.943 | Uniform(0.75,1.00) | Brisson^7^ |
| UTI/ PA | 0.711 | Uniform(0.57,0.85) | Whiting^4^ |
| Non UTI Illness | 0.943 | Uniform(0.75,1.00) | Brisson^7^ |
| No ESRD (Well Adult) | 1.000 | Fixed | NA |
| Dialysis | 0.430 | Uniform(0.34,0.52) | Churchill^8^ |
| Transplant | 0.840 | Uniform(0.67,1.00) | Churchill^8^ |

Supplemental table 5: Comparison of mean TAPQOL domain scores by GP Diagnosis^[[14]](#footnote-14)^

| **Domain** | **UTI (N=58)** | **URTI (N=229)** | **Viral Illness (N=109)** | **Otitis Media (N=66)** | **Chest Infection (N=46)** | **Tonsillitis (N=29)** | **Gastroenteritis (N=26)** | **Other (N=112)** |
| --- | --- | --- | --- | --- | --- | --- | --- | --- |
| Sleeping | 0.598 | 0.569 | 0.592 | 0.538 | 0.511 | 0.504 | 0.603 | 0.611 |
| Appetite | 0.603 | 0.654 | 0.667 | 0.667 | 0.607 | 0.526 | 0.564 | 0.683 |
| Lungs | 0.954 | 0.912 | 0.939 | 0.962 | 0.79 | 0.848 | 0.978 | 0.921 |
| Stomach | 0.803 | 0.870 | 0.818 | 0.889 | 0.855 | 0.802 | 0.638 | 0.804 |
| Skin | 0.909 | 0.875 | 0.896 | 0.876 | 0.909 | 0.888 | 0.933 | 0.878 |
| Motor | 0.948 | 0.939 | 0.952 | 0.950 | 0.924 | 0.881 | 0.910 | 0.942 |
| Social | 0.841 | 0.799 | 0.817 | 0.795 | 0.819 | 0.795 | 0.796 | 0.795 |
| Problem | 0.741 | 0.689 | 0.706 | 0.697 | 0.685 | 0.653 | 0.676 | 0.698 |
| Communication | 0.949 | 0.944 | 0.971 | 0.909 | 0.952 | 0.847 | 0.958 | 0.933 |
| Anxiety | 0.845 | 0.877 | 0.867 | 0.889 | 0.830 | 0.902 | 0.859 | 0.823 |
| Positive | 0.739 | 0.750 | 0.752 | 0.750 | 0.685 | 0.707 | 0.699 | 0.733 |
| Liveliness | 0.615 | 0.702 | 0.734 | 0.727 | 0.645 | 0.701 | 0.590 | 0.687 |

Supplemental table 6: Sensitivity analysis, short-term costs and outcomes^[[15]](#footnote-15)^

|  | UTI Prevalence | | Lab Diagnostic Accuracy | | ABX Treatment Effect | | UTI Utility | | Simple | |
| --- | --- | --- | --- | --- | --- | --- | --- | --- | --- | --- |
|  | **CJ** | **DUTY5%** | **CJ** | **DUTY5%** | **CJ** | **DUTY5%** | **CJ** | **DUTY5%** | **CJ** | **DUTY5%** |
| ***Diagnostic pathway*** | |  |  |  |  |  |  |  |  |  |
| Urine sample requested (%) | 12.64 | 8.80 | 9.12 | 4.79 | 9.12 | 4.79 | 9.12 | 4.79 | 9.12 | 4.79 |
| Sensitivity – urine sampling | 0.564 | 0.582 | 0.564 | 0.582 | 0.564 | 0.582 | 0.564 | 0.582 | 0.564 | 0.582 |
| Specificity – urine sampling | 0.915 | 0.962 | 0.915 | 0.962 | 0.915 | 0.962 | 0.915 | 0.962 | 0.915 | 0.962 |
| Sensitivity – after laboratory test | 0.426 | 0.439 | 0.539 | 0.557 | 0.426 | 0.439 | 0.426 | 0.439 | 0.426 | 0.439 |
| Specificity – after laboratory test | 0.998 | 0.999 | 1.000 | 1.000 | 0.998 | 0.999 | 0.998 | 0.999 | 0.998 | 0.999 |
| ***Treatment pathway (children with UTI)*** |  |  |  |  |  |  |  |  |  |  |
| Immediate, appropriate^[[16]](#footnote-16)^ antibiotic (%) | 36.64 | 34.05 | 37.06 | 34.42 | 36.64 | 34.05 | 36.64 | 34.05 | 36.64 | 34.05 |
| Laboratory informed^[[17]](#footnote-17)^, appropriate antibiotic (%) | 12.51 | 16.55 | 15.21 | 20.14 | 12.51 | 16.55 | 12.51 | 16.55 | 12.51 | 16.55 |
| Inappropriate antibiotic (%) | 17.56 | 16.50 | 16.65 | 15.46 | 17.56 | 16.50 | 17.56 | 16.50 | 17.56 | 16.50 |
| No antibiotic (%) | 33.29 | 32.90 | 31.09 | 29.97 | 33.29 | 32.90 | 33.29 | 32.90 | 33.29 | 32.90 |
| ***Treatment pathway (children without UTI)*** |  |  |  |  |  |  |  |  |  |  |
| Antibiotic treatment for UTI (%) | 4.79 | 1.62 | 4.71 | 1.57 | 4.79 | 1.62 | 4.79 | 1.62 | 4.786 | 1.616 |
| ***Short term costs and outcomes*** |  |  |  |  |  |  |  |  |  |  |
| **Costs per child** |  |  |  |  |  |  |  |  |  |  |
| Sampling, culture, antibiotic treatment costs | 2.74 | 2.04 | 2.00 | 1.22 | 1.99 | 1.22 | 1.99 | 1.22 | 1.99 | 1.22 |
| Initial (21 day) health service costs | 46.65 | 45.95 | 44.05 | 43.27 | 43.99 | 43.21 | 44.06 | 43.28 | 43.94 | 43.17 |
| **Outcomes** |  |  |  |  |  |  |  |  |  |  |
| Asymptomatic days | 16.40 | 16.40 | 16.35 | 16.35 | 16.35 | 16.35 | 16.34 | 16.34 | 16.34 | 16.34 |
| Short term average QALDs^[[18]](#footnote-18)^ | 20.72 | 20.72 | 20.73 | 20.73 | 20.73 | 20.73 | 20.73 | 20.73 | 20.73 | 20.73 |
| **Cost-effectiveness** |  |  |  |  |  |  |  |  |  |  |
| iNMB^[[19]](#footnote-19)^ per child | --- | 0.70 | --- | 0.77 | --- | 0.78 | --- | 0.78 | --- | 0.78 |

Supplemental table 7: Sensitivity analysis, medium and long-term costs and outcomes^[[20]](#footnote-20)^

|  | **ESRD Probability** | | **PRS Probability** | |
| --- | --- | --- | --- | --- |
|  | **CJ** | **DUTY5%** | **CJ** | **DUTY5%** |
| Average number UTI recurrence at 3 years / 10,000 patients | 165.5 | 165.5 | 165.5 | 165.5 |
| % End Stage Renal Disease | 0.477 | 0.477 | 0.501 | 0.501 |
| Average years lived | 72.84 | 72.84 | 72.83 | 72.83 |
| Average Lifetime Cost | 303.1 | 300.7 | 315.8 | 313.5 |
| Average Lifetime QALYs | 25.66 | 25.66 | 25.65 | 25.65 |
| iNMB^[[21]](#footnote-21)^, per child | --- | 2.28 | --- | 2.28 |

**References**

1. Lord Carter of Coles. Report of the second phase of the review of NHS pathology services in England 2008.

2. The Information Centre. Prescription Cost Analysis: England, 2011.

3. Department of Health. 2010-11 Reference Costs 2011.

4. Whiting P, Westwood M, Bojke L, et al. Clinical effectiveness and cost-effectiveness of tests for the diagnosis and investigation of urinary tract infection in children: a systematic review and economic model. Health Technol Assess 2006;10(36):iii-iv, xi-xiii, 1-154.

5. Nagler EV, Williams G, Hodson EM, et al. Interventions for primary vesicoureteric reflux. Cochrane Database Syst Rev 2011(6):CD001532.

6. Baboolal K, McEwan P, Sondhi S, et al. The cost of renal dialysis in a UK setting--a multicentre study. Nephrol Dial Transplant 2008;23(6):1982-9.

7. Brisson M, Senecal M, Drolet M, et al. Health-related quality of life lost to rotavirus-associated gastroenteritis in children and their parents: a Canadian prospective study. Pediatr Infect Dis J 2010;29(1):73-5.

8. Churchill DN, Torrance GW, Taylor DW, et al. Measurement of quality of life in end-stage renal disease: the time trade-off approach. Clin Invest Med 1987;10(1):14-20.

1. UTI: urinary tract infection; CI: confidence interval; ref: reference group [↑](#footnote-ref-1)
2. UTI: urinary tract infection. The points-based model was designed to be simple to apply and excluded the two variables (abdominal tenderness and absence of ear abnormalities) that contributed least to the predictive accuracy of the statistical model. [↑](#footnote-ref-2)
3. Cut-points represent the probability of urinary tract infection predicted by DUTY coefficient-based algorithm [↑](#footnote-ref-3)
4. Cut-points represent the score in DUTY points-based algorithm [↑](#footnote-ref-4)
5. UTI: urinary tract infection; PA: pyelonephritic attack [↑](#footnote-ref-5)
6. This is a simplification of the actual model which included separate states depending on how quickly the child received an antibiotic (delayed vs. immediate) and if the uropathogen was sensitive to the prescribed treatment (sensitive vs. resistant) [↑](#footnote-ref-6)
7. UTI: urinary tract infection; PA: pyelonephritic attack. Figure represents the movement of patient within the medium term model in each year of the model. UTIs are more likely for patients with VUR although part of this increase is mitigated for patients with treated VUR. Patients may move between the untreated VUR and treated VUR if they receive a correct VUR diagnosis. [↑](#footnote-ref-7)
8. UTI: urinary tract infection; PA: pyelonephritic attack; VUR: vesicoureteral reflux [↑](#footnote-ref-8)
9. PA: pyelonephritic attack; PRS: progressive renal scarring; ESRD: end-stage renal disease [↑](#footnote-ref-9)
10. We used responses to the question ‘How many days since [name of child] joined the DUTY study (day 0) was it until [his/her] symptoms improved?’ in the 14 day questionnaire data to calculate symptom duration. A small proportion of parents of children both with and without UTI reported symptom recovery times greater than 14 days hence we extrapolated our estimates to 21 days by which time the vast majority of children were predicted to have become asymptomatic. We fitted Weibull models (shape = 1.487, scale = 0.096) for children with UTI and (shape = 1.270, scale = 0.1016) for those without. [↑](#footnote-ref-10)
11. GP: general practitioner; MCUG: micturating cystourethrogram; UTI: urinary tract infection; PA: pyelonephritic attack; ESRD: end-stage renal disease [↑](#footnote-ref-11)
12. All costs were modelled as uniform distributions with lower and upper bounds -/+50% of the mean. All utilities were modelled as uniform distributions with lower and upper bounds -/+20% of the mean. [↑](#footnote-ref-12)
13. Assuming healthcare resource group LA02A. [↑](#footnote-ref-13)
14. UTI: urinary tract infection; URTI: upper respiratory tract infection. Diseases are based on the working diagnosis of the consulting GP except for UTI where only those with laboratory confirmed UTI are included [↑](#footnote-ref-14)
15. CJ: clinical judgement; UTI: urinary tract infection; QALDs: quality-adjusted life days; iNMB: incremental net monetary benefit [↑](#footnote-ref-15)
16. (In)appropriate defined as an antibiotic to which the bacterium is (not) sensitive. [↑](#footnote-ref-16)
17. Antibiotic prescribing determined by laboratory result, usually started a few days after primary care attendance. [↑](#footnote-ref-17)
18. [↑](#footnote-ref-18)
19. Based on a £20,000 per QALY threshold; compared to clinical judgement; a positive value indicates that the strategy is more cost-effective than clinical judgement [↑](#footnote-ref-19)
20. CJ: clinical judgement; UTI: urinary tract infection; ESRD: end-stage renal disease; QALYs: quality-adjusted life years; iNMB: Incremental net monetary benefit [↑](#footnote-ref-20)
21. Based on a £20,000 per QALY threshold; compared to clinical judgement strategy; a positive value indicates that the strategy is more cost-effective than clinical judgement [↑](#footnote-ref-21)
